# A multi-level meta-analysis of the relationship between decision-making during birth and postpartum mental health

**DOI:** 10.1080/21642850.2025.2456032

**Published:** 2025-02-04

**Authors:** Louisa Arnold, Marie Völkel, Jenny Rosendahl, Michael Rost

**Affiliations:** a Department for Health Psychology, FernUniversität in Hagen, Hagen, Germany; b Wilhelm Wundt Institute for Psychology, University of Leipzig, Leipzig, Germany; c Institute of Psychosocial Medicine, Psychotherapy and Psychooncology, Jena University Hospital, Jena, Germany; d Institute for Biomedical Ethics, University of Basel, Basel, Switzerland

**Keywords:** Birth, decision-making, depression, post-traumatic stress disorder, meta-analysis, moderator, ethics

## Abstract

**Introduction:**

There is accumulating evidence of ineffective decision-making between birthing individuals and healthcare providers during childbirth. While research syntheses have demonstrated that negative birth experiences are associated with postpartum mental health, primary quantitative studies linking specific decision-making measures and mental health outcomes have not been synthesised. The present study aims to fill this gap in order to provide hands-on evidence on how to further improve perinatal care.

**Methods:**

A systematic literature search using Bolean logic was conducted. A final set of 34 publications from 14 different countries could be included in our meta-analysis. Measures of intrapartum decision-making were consolidated into four key domains: information, respect, control, and involvement. We conducted multi-level meta-analyses to assess the overall relationship of intra-partum decision-making and mental-health outcomes, as well as the specific correlations associated with each decision-making domain.

**Results:**

Our analysisrevealed that less effective intrapartum decision-making is associated with more postpartum overall mental health problems (*r* = -.25), depression (*r* = -.19), and posttraumatic stress disorder (*r* = -.29). More precisely, while all domains of intrapartum decision-making (information: *r* = -.22, involvement: *r* = -.23, respect: *r* = -.28, control: *r* = -.25) were associated with postpartum overall psychopathology, only information (*r* = -.18), respect (*r* = -.25), and control (*r* = -.12) were associated with depression, and only involvement (*r* = -.31), respect (*r* = -.32), and control (*r* = -.25) were associated with posttraumatic stress disorder. A higher percentange of planned cesarean sections in a sample and longer time lags between birth and post-effect measurement were identified as moderating variables.

**Conclusions:**

Ineffective decision-making is a significant contributing factor to the development of adverse postpartum mental health problems outcomes. Implications for practice concern establishing numerous antenatal care contacts as a standard to enhance birth preparedness for both birthing individuals and providers. Additionally, measuring the experience of intrapartum decision-making as an indicator of quality of care as a default to monitor, analyse, and improve decision-making and to facilitate accountability systems.

## Introduction

Globally, there is accumulating evidence of birthing people’s negative birth experiences owing to poor interactions with providers, with many of them being directly related to the decision-making process between the two parties; for example, disrespectful care or insufficient informed consent (Ansari & Yeravdekar, 2020; Bohren et al., 2019; Niles et al., 2021; Oelhafen et al., 2020; van der Pijl et al., 2020; Vedam et al., 2019). In this sense, ineffective decision-making does not refer to the ‘what’ of decision-making (e.g. the issue at hand, decisions being made), but the ‘how’ of decision-making at the interpersonal level, that is whether a birthing person’s rights are being respected. According to the World Health Organization (WHO), instances of ineffective decision-making (e.g. loss of autonomy, dismissing birthing people’s concerns, lack of respect for birthing people’s preferred birth position) qualify as mistreatment in birth (Bohren et al., 2015), and, as such, represent human rights violations (World Health Organization, 2014). They are antithetical to quality care (World Health Organization, 2016), and cause adverse psychological outcomes on the part of birthing people (Dekel et al., 2017; Oelhafen et al., 2021), ultimately affecting mother–child-bonding and child development (Cook et al., 2018; Suetsugu et al., 2020) as well as parental couple relationships (Garthus-Niegel et al., 2018; Parfitt & Ayers, 2009). Furthermore, they result in a decreased likelihood of giving birth again (Gottvall & Waldenström, 2002; Størksen et al., 2013), and of accessing a birth facility for birth (Mselle et al., 2013; Silan et al., 2014).

During decision-making in birth birthing people, their companions, and providers actively work together to make birth-related decisions (e.g. interventions). It has been argued that a shift from shared to person-centred decision-making is needed to overcome inherent power differentials and to prioritise birthing people’s preferences over providers’ (Rost et al., 2022; Rost et al., 2023; Vedam et al., 2019). Conceptually, the imperative to centre birthing people in intrapartum care to ultimately improve reproductive health encompasses eight domains: dignity, autonomy, privacy, communication, social support, supportive care, trust, and the health facility environment (Sudhinaraset et al., 2017). Such conceptual frameworks have been translated into tangible multi-step models of person-centred decision-making in birth (Birth Place Lab, 2022).

A plethora of studies have been devoted to postpartum adverse psychological outcomes, with the majority focusing on postpartum depression (PP-D) and postpartum posttraumatic stress disorder (PP-PTSD) (Batt et al., 2020; Grekin & O'Hara, 2014). Available evidence points towards poor shared decision-making (Shay & Lafata, 2015), low levels of perceived control (DeLuca & Lobel, 2014), and verbal violence and negligence (Souza et al., 2017) as predictors of PP-D; towards negative subjective birth experiences (Ayers et al., 2016; Garthus-Niegel et al., 2013), lack of support (Ayers et al., 2016) – especially for birthing people with trauma histories (Ford, 2011), low satisfaction with providers (Dikmen Yildiz et al., 2017), poor quality of interactions with providers (Simpson & Catling, 2016), feelings of powerlessness (Menage, 1993), and lack of control (Harris & Ayers, 2012; Olde et al., 2006) as predictors of PP-PTSD.

While existing research syntheses have demonstrated that negative birth experiences (e.g. obstetric violence, negative delivery experience) are associated with adverse psychological outcomes (e.g. PP-PTSD, PP-D) (Ayers et al., 2016; Dekel et al., 2017), the specific impact of decision-making in birth as one key determinant of subjective birth experiences has not been examined in these studies. Hence, available primary quantitative studies assessing the association between hands-on measures of intrapartum decision-making and postpartum mental health outcomes have not been systematically synthesised, yet. Hence, the objective of our multi-level meta-analysis was to examine the effects of decision-making in birth on postpartum mental health. Since decision-making is primarily enabled by providers and health systems, it is a major modifiable component in the genesis of postpartum psychopathology and, as such, a suitable candidate to effectively improve birth experiences. By illuminating the various pathways through which aspects of decision-making in birth (e.g. information, involvement) can translate into adverse psychological outcomes, our findings can serve as tangible points of leverage for prevention, for example through provider education. Lastly, they serve a dual purpose: they improve reproductive health and reproductive rights.

## Materials and methods

### Search strategy and information sources

We followed the Preferred Reporting Items for Systematic Reviews and Meta-Analyses (PRISMA) (Page et al., 2021). We used Boolean logic and four search blocks to capture the two concepts of interest and the two respective time periods, that is (1) decision-making that (2) occurs in birth, and (3) psychological outcomes that (4) occur after birth (Appendix Table A1). We searched Scopus, PubMed, PsycInfo, Web of Science, Cinahl, and SocIndex (Appendix Table A1). Date of last search was 16/12/2021. We contacted authors to obtain unreported data, screened references of included articles for relevant studies, attempted to solicit additional citations through topic experts, and managed references through EndNote.

### Eligibility criteria, selection process, and search results

We defined the following eligibility criteria: primary studies had to (a) be quantitative, (b) include a measurement of (in-)effective decision-making (studies using scales on the birth experience that only partially measured decision-making variables were considered eligible, if they consisted of at least fifty-percent items measuring decision-making.) and (c) one psychological outcome, (d) report a statistical information on the relationship between the measure of decision-making and psychological outcomes (i.e. correlations, simple linear regression coefficients, odds ratios, contingency tables, means and standard deviations, t-tests; if none were available, but conversion methods allowed estimation of the relationship, studies were included), and (e) be written in English, French, German, or Spanish. No limitations were placed on whether the study was peer-reviewed, study design, and publication date.

**Figure 1.**
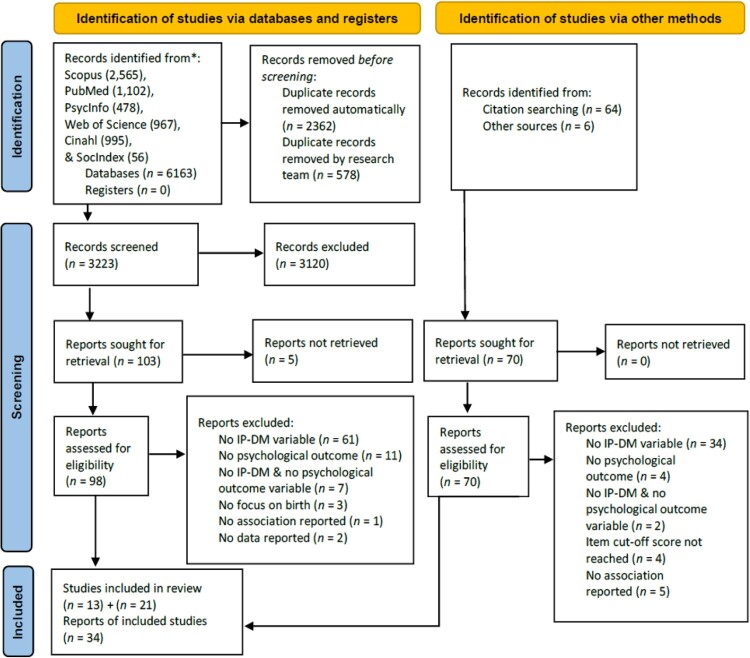
PRISMA flowchart for inclusion of studies.

Search resulted in 6.163 studies. After de-duplication, 3.223 remained. Two researchers independently reviewed titles and abstracts. This step resulted in 103 articles selected for full-text review; however, five articles could not be retrieved. Besides, 70 articles were chosen from other sources; namely through citation searching (World Health Organization, 2015) and based on the research team’s knowledge (Ansari & Yeravdekar, 2020). After full-text review 34 articles remained (Figure 1). At any point of the screening process uncertainties were solved through discussions.

### Data coding

We developed a 210-item codebook covering the following main areas of interest: description of publications (e.g. year, country), study design (e.g. longitudinal, cross-sectional), sample characteristics (e.g. sample size, age), intrapartum situation (e.g. birth setting, birth mode), decision-making variables (e.g. being informed, control), psychological outcomes (e.g. PP-D, PP-PTSD), and statistics (e.g. means, correlations; if no correlation was reported, effect sizes were transformed into correlations (Rosenthal et al., 1994)). If studies reported more than one effect for the exact same relationship between a dimension of decision-making and a specific outcome, effects were averaged. If necessary, calculated effect sizes were recoded to ensure that a negative correlation always represented a negative psychological outcome following ineffective decision-making. MV carried out the coding, LA checked accuracy and completeness on a 15% sample, and disagreements were discussed in the research team.

### Grouping of measurement tools and investigated associations

The measurement tools and their items were compared with one another. Recurring themes were collected, discussed, and ultimately harmonised. For this purpose, we considered both the measurement tools’ original labelling and existing models of intrapartum decision-making (Birth Place Lab, 2022). This process resulted in the following four domains of decision-making in birth: being informed, being involved, being respected, and having control (see Appendix Table A2 for more information on the included items and scales). Scales and items measuring being informed contain both the provision and the quality of information. Being involved was measured via scales and items that referred to the dyad of birthing person and provider and captured aspects of their interaction related to the participation of the birthing person. Scales and items measuring being respected captured the behavior of providers both regarding the person’s circumstances, identity, and dignity and the person’s decisions and preferences. The last group, having control, captures the birthing persons’ sense of control over the decision-making.

The vast majority of studies reported PP-D and PP-PTSD as mental health outcomes, only few other measures were included in the composite main effect. A range of established questionnaires were used to measure outcomes. For PP-D, the most frequently employed tool was the Edinburgh Postnatal Depression Scale (*k* = 8), while for PP-PTSD, the most commonly used tool was the Impact of Event Scale-Revised (*k* = 4).

### Quality appraisal and interrater agreement

We used the Effective Public Health Practice Project Quality Assessment Tool for Quantitative Studies (Table 1) (Thomas et al., 1998 ). We adapted the tool as studies of interest were not intervention studies. Five studies were randomly selected and assessed for methodological quality by two coders independently. Studies were rated as ‘strong’, ‘moderate’ or ‘weak’ in each domain. Cohen’s kappa was calculated to assess interrater reliability for the tool’s six domains and the overall quality rating. A weighted Kappa of .726 indicated substantial agreement (Viera & Garrett, 2005).

**Table 1.** Included studies

Authors	Year	*N*	Country	Design	Quality
Astbury et al.	1994	771	Australia	Cross-sectional	Weak
Avignon et al. (Avignon et al.) (Astbury et al., 1994; Astbury et al., 1994; Astbury et al., 1994; Astbury et al., 1994; Rost et al., 2020; Rost et al., 2020; Rost et al., 2020)	2021	794	Switzerland	Cross-sectional	Weak
Ayers et al.	2014	76	UK	Longitudinal (cross-sectional data used)	Moderate
Beck et al.	2011	1 573	USA	Longitudinal only post-birth	Weak
Creedy et al.	2000	592	Australia	Longitudinal (cross-sectional data used)	Weak
Czarnocka & Slade	2000	298	UK	Longitudinal only post-birth	Strong
De Schepper et al.	2016	340	Belgium	Longitudinal only post-birth	Moderate
DeLuca & Lobel	2014	240	USA	Longitudinal (cross-sectional data used)	Weak
Denis et al.	2011	239	France	Longitudinal only post-birth	Moderate
Fair & Morrison	2012	33	USA	Longitudinal (cross-sectional data used)	Weak
Ford & Ayers	2011	138	UK	Longitudinal pre – and post-birth	Moderate
Gökçe İsbİr et al.	2016	270	Turkey	Longitudinal (cross-sectional data used)	Strong
Green & Baston	2003	425	UK	Longitudinal (cross-sectional data used)	Moderate
	2003	564	UK	Longitudinal (cross-sectional data used)	Moderate
Green et al.	1990	825	UK	Longitudinal (cross-sectional data used)	Moderate
Henderson & Redshaw	2013	5 333	UK	Cross-sectional	Weak
Keogh et al.	2002	42	UK	Longitudinal (cross-sectional data used)	Moderate
King et al.	2017	157	UK	Cross-sectional	Weak
Kjerulff et al.	2021	3 080	USA	Longitudinal (cross-sectional data used)	Weak
Kountanis et al.	2021	600	USA	Longitudinal pre – and post-birth	Moderate
Leeds & Hargreaves	2008	102	UK	Cross-sectional	Weak
Limmer et al.	2021	2 045	Germany	Cross-sectional	Weak
Martinez-Vázquez et al.	2021	839	Spain	Cross-sectional	Weak
Michels et al.	2013	664	Australia	Cross-sectional	Weak
Mohammad et al.	2011	353	Jordan	Longitudinal pre – and post-birth	Moderate
Oelhafen et al.	2021	6 054	Switzerland	Cross-sectional	Weak
Redshaw & Henderson	2013	5 332	UK	Cross-sectional	Weak
Small et al.	2003	318	Australia	Cross-sectional	Weak
Soet et al.	2003	112	USA	Longitudinal pre – and post-birth	Moderate
Sorenson & Tschetter	2010	71	USA	Cross-sectional	Weak
Souza et al.	2017	432	Brazil	Cross-sectional	Weak
Stevens et al.	2012	187	USA	Cross-sectional	Weak
Sudhinaraset et al.	2021	1 014	Kenya	Longitudinal only post-birth	Moderate
Tomsis et al.	2021	306	Israel	Longitudinal only post-birth	Weak
Verreault et al.	2012	367	Canada	Longitudinal pre – and post-birth	Weak

Note*:* Green and Baston (2003) report separate statistical analyses for primiparas and multiparas; thus, the publication appears twice. In our here presented multi-level-analyes the study will be included as one study on the publication-level and as two separate samples on the study – and effect-size level.

### Synthesis methods

**Figure 2.**
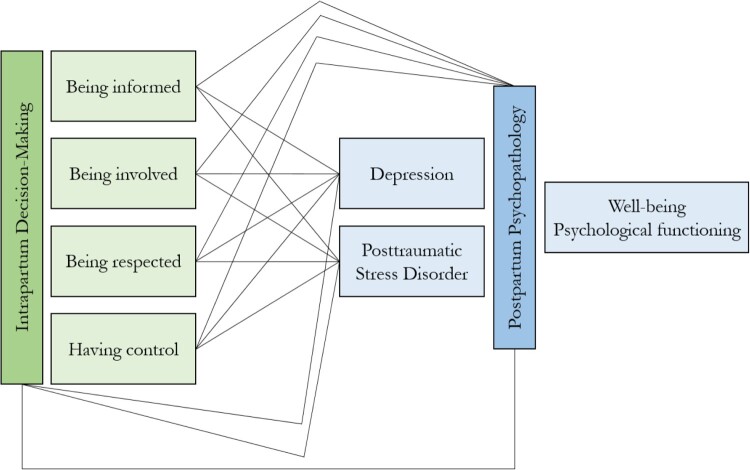
Investigated relationships.

All analyses were performed in *R* 4.0.3 using the metafor package and dmetar package (Harrer et al., 2019; Viechtbauer, 2010). Since most studies reported more than one effect size of interest, dependence between the effect sizes was very likely. To account for this a random-effects multi-level model with Knapp-Hartung adjustments (IntHout et al., 2014; Sidik & Jonkman, 2005) was fitted with a level for sampling variance (Level 1), one for the variance among the effect sizes within the studies (Level 2), and one level for the between-study variance (Level 3). Model parameters were estimated using a restricted maximum likelihood estimation (Assink & Wibbelink, 2016). Besides pooling across all studies to estimate the overall mean effect sizes of the association, effect sizes were also pooled based on the outcome measure (PP-D and PP-PTSD) as well as the different dimensions of decision-making (Figure 2). To investigate whether the three-level model was well suited to describe the data, log-likelihood ratio tests were performed testing whether constraining variances on level 2 and level 3 to zero respectively, significantly deteriorated parameters of model fit.

Heterogeneity of the effect sizes was quantified using *I^2^* (Higgins & Thompson, 2002) and explored on each level of the model (Cheung, 2014). An *I^2^* of 25% is regarded as low, 50% as moderate and 75% or more as a substantial amount of heterogeneity (Higgins et al., 2003). Furthermore, the test of the coefficient *Q* indicates whether there is significant heterogeneity in the whole model. Moderators both on Level 2 and 3 were tested with an Omnibus test indicating whether these moderators significantly reduced heterogeneity. Since tests of publication bias have not been validated for multi-level meta-analyses yet (Assink & Wibbelink, 2016), the common tests for missing data have been performed on the univariate model (Duval & Tweedie, 2000; Egger et al., 1997).

## Ethics statement

The study was conducted in accordance with the Declaration of Helsinki and was evaluated by an Institutional Review Board/Ethics committee. The study received an exemption from an Institutional Review Board/Ethics committee.

## Results

### Study pool

Included publications (*k *= 34; Table 1) were published between 1990 and 2021. Most studies were published in a peer-reviewed journal (*k *= 33, 97.1%) and conducted in high-income countries (*k* = 29, 85.3%; UK (*k* = 10, 29.4%); US/Canada (*k* = 8, 26.5%)) (World Bank, 2022).

In total, *N *= 34.586 birthing people participated. Average sample size was 988.17 (*SD *= 1552.26; *Min* = 33; *Max* = 6054.).

Concerning sample characteristics the synthesis of primary studies in this meta-analysis reveals a significant variance in the extent of information provided across the included samples, resulting in an aggregated portrayal of the studied populations that exhibits notable gaps. In certain instances, the prevalence of specific characteristics is expounded upon by only a limited number of studies, consequently imparting a lack of comprehensive representation to the overall collection of research. Consequently it is imperative to consider the number of studies *k* contributing information pertaining to specific sample attributes. Across all included samples the mean age was 30 years (*SD *= 2.15, *k* = 34; one study did not report the age of the sample). In most studies, information on the etnhic composition of the samples was not reported (*k *= 23). However, in 48.6% of the samples that provided the respective information (*k* = 17), the largest ethnic group was Caucasian, while the remaining samples where this information was available were more diverse (*k* = 3). Based on information available in *k* = 18 studies, on average, 43.1% of birthing people had a high school degree or less as the highest educational level and 9.2% were single (based on *k* = 24). The proportion of birthing people with prenatally existing psychological vulnerability were as follows: depression (*k *= 5; 14.3%), acute PTSD (*k *= 1; 2.9%), general psychological distress (*k *= 3; 8.6%), consulting a psychologist or psychiatrist at least once in the past (*k *= 1, 2.9%), previous traumatic event (*k *= 3, 8.6%), history of anxiety or depression (*k *= 1, 2.9%). The mean percentage of primiparous birthing people was 57.9% (*k *= 29). The samples consisted mostly of hospital-births (98.2%, based on *k* = 22), carried out as vaginal non-instrumental births (61.4%), as unplanned c-section, or instrumental births (e.g. forceps or ventouse; 32.4%, *k* = 18), or planned c-section (8.9%, *k* = 21).

In 24 studies (70.6%) the variables of interest were measured cross-sectionally after birth. Except for one study, the postpartum psychological outcomes were assessed with standardised questionnaires (Callahan et al., 2006; Cox et al., 1987). The measures of decision-making in birth were entirely assessed with standardised tools (Ford & Wright, 2009; Vedam et al., 2017), but most tools where rather broad, covering a wider range of birth experiences. Consequently, for the meta-analysis subscales or responses to single items specifically focusing on decision-making were selected (Appendix Table A2).

### Main effects

First, an overall effect for the association between decision-making and postpartum mental health was computed. Based on a multi-level model, a significant overall effect size of *r* = −0.25 (SE = 0.0242, *p* < 0.0001) was estimated, indicating that less effective decision-making goes along with more adverse psychological outcomes. Primary studies’ correlations ranged from *r* = −0.69 to *r* = 0.20 with only two effect sizes being larger or equal to zero. No statistical outliers were present.

As a second step, multi-level models were computed separately for the two main outcome measures: PP-D and PP-PTSD (Table 2). A small number of effect sizes described the correlations between decision-making and well-being (*k* = 4) and psychological functioning (*k* = 1). They were inversed so that effects described correlations between decision-making and adverse psychological outcomes and pooled for an average effect (‘Other’ in Table 2).

**Table 2.** Correlations between intrapartum decision-making and postpartum mental health.

Psychological outcome	*r*	SE	95% CI	Total *I^2^*	*k*	*N*(ES)
Overall Mental Health	−0.25***	0.0242	−0.29; – 0.20	94.1%***	35	55
PP-D	−0.19***	0.0351	−0.27; – 0.12	91.9%***	13	18
PP-PTSD	−0.29***	0.0317	−0.35; – 0.22	92.3%***	21	30
Other	−0.20*	0.0617	−0.35; – 0.05	94.2%***	5	7

Note: *p < 0.05, **p < 0.01, ***p < 0.0001. *I^2^* = percentage of variability across all levels that is not due to sampling error. *k* = number of independent studies. *N*(ES) = number of effect sizes. Other = well-being and psychological functioning.

In a third step, measures of decision-making were dissected more precisely into the different domains (information, involvement, respect, and control) and regarded with respect to overall mental health as well as the two outcome groups, PP-D and PP-PTSD. Concerning overall mental health, analyses showed that all four domains of decision-making were significantly related to the outcome variables (Table 3).

**Table 3.** Correlations between the four domains of intrapartum decision-making and postpartum mental health.

	*r*	SE	95% CI	Total *I^2^*	*k*	*N* ES
**Overall Mental Health**						
Information	−0.22**	0.0472	−0.34; – 0.10	82.2%***	6	7
Involvement	−0.23***	0.0481	−0.33; – 0.12	97.2%***	15	18
Respect	−0.28***	0.0436	−0.37; – 0.19	96.6%***	14	14
Control	−0.25***	0.0267	−0.30; – 0.19	56.4%**	14	16
**PP-D**						
Information	−0.18*	0.0377	−0.35; – 0.03	53.7%	3	3
Involvement	−0.14	0.1013	−0.42; – 0.15	94.1%***	5	5
Respect	−0.25*	0.0659	−0.43; – 0.07	95.8%***	5	5
Control	−0.18**	0.0336	−0.27; – 0.09	32.9%	5	5
**PP-PTSD**						
Information	−0.37	0.0858	−0.15; – 0.72	50.5%	2	2
Involvement	−0.31***	0.0591	−0.44; – 0.17	96.3%***	10	10
Respect	−0.32**	0.0637	−0.47; – 0.16	95.5%***	8	8
Control	−0.25***	0.0238	−0.31; – 0.20	0%	10	10

Note: *p < 0.05, **p < 0.01, ***p < 0.0001. *I^2^* = percentage of variability across all levels that is not due to sampling error. *k* = number of independent studies. *N* ES = Number of effect sizes.

For PP-D, analyses revealed significant correlations with information, respect, and control (not with involvement). For PP-PTSD, analyses revealed significant correlations with involvement, respect, and control (not with information). It has to be taken into account, that neither of these analyses were based on more than 10 studies, thus testing for significance is weary and the results have to be regarded with much caution. Effect sizes for PP-PTSD were on average higher than for PP-D, the confidence intervals, however, overlapped, indicating that the difference was not significant.

### Model fit and variance distributions

For the main model concerning overall mental health and the two psychological outcomes, PP-D and PP-PTSD, the proportions of variance explained by other than sampling variance were explored. Also, a comparison of the multi-level model with a more constrained model was carried out to determine whether the multi-level-model was indeed the best fit for the data. To do so, the Level 2 variance, which is the variance of the effect sizes within the studies, was set to zero. The likelihood ratio test comparing the restricted with the full model was significant (χ^2^ [1] = 43.9, *p* < 0.0001) favouring the latter. Setting the Level 3 variance, the between-study heterogeneity, to zero is equal to fitting a simple random effects model in which the independence of all effect sizes is assumed. Comparing this model to the three-level-model also pointed towards accepting the full model (χ^2^ [1] = 8.7, *p* = 0.003). The same procedures were also carried out for the subset of correlations with PP-D (Level 2 heterogeneity removed: χ^2^ [1] = 17.4, *p* < 0.0001; Level 3 heterogeneity removed: χ^2^ [1] = 0.0, *p* = 0.859) and PP-PTSD (Level 2 heterogeneity removed: χ^2^ [1] = 14.1, *p* = 0.000; Level 3 heterogeneity removed: χ^2^ [1] = 11.4, *p* = 0.001) separately. While for PP-D outcomes the results were mixed but nevertheless pointing towards a multi-level approach, for PP-PTSD outcomes the model comparisons clearly favoured the multi-level-model. Thus, in all further analyses the nested data structure will be modelled to estimate the pooled effects.

For the three models (overall, PP-D, PP-PTSD) the percentage of total variance attributable to each of the levels was explored to locate heterogeneity. For the overall model, the sampling error variance on Level 1 was *I^2^* = 5.9%. On Level 2 the amount of variance explained by other than sampling variance (heterogeneity within the studies) was *I^2^* = 38.9% and on Level 3 (between-study variance) heterogeneity made up *I^2^* = 55.2%. Thus, across all levels *I^2^* = 94.1% of the variance was not attributable to sampling error calling for subsequent moderator analyses, which was further supported by a negative Q-Statistic (Q[54] = 2284.631; *p* < 0.0001). For PP-D correlations the amount of heterogeneity on Level 2 stood out (Level 1; 8.1%; Level 2: 84.1%; Level 3: 7.8%, total: 91.9%), whereas for PP-PTSD the largest amount of heterogeneity was located in the between-study variability (Level 1: 7.7%; Level 2: 9.5%; Level 3: 82.8%; total 92.3%). For PP-D outcomes, the overall test for heterogeneity was significant (Q[17] = 717.899; *p* < 0.0001) as well as for the PP-PTSD correlations (Q[29] = 935.480; *p* < 0.0001).

### Moderator analyses

Since there was significant heterogeneity on both level 1 and 2, moderator analyses were conducted to explore the sources of variance between the studies and between the effect sizes within the studies. On the study-level characteristics of the sample and the study design were tested, on the effect size level characteristics of measurement.

First, moderators were tested with regard to overall mental health (Table 4). Among the tested variables only one proved to be a significant moderator: A higher percentage of birthing people in the sample who had a planned c-section was related to stronger negative correlations between decision making and psychopathology (*b* = −0.008, *p* = 0.011, F[1, 31] = 7.300, *p* = 0.011, QE[31] = 585.345, *p* < 0.0001, *k* = 21, *N* ES = 33). This effect could be demonstrated even more clearly when comparing samples with less than 15% planned c-sections and those with more than that: in the former the average effect was *r* = −0.201 while for the latter the average effect was *r* = −0.468 (*b* = −0.267, *p* < 0.0001, F[1, 31] = 22.469, *p* < 0.0001, QE[31] = 259.211, *p* < 0.0001).

**Table 4.** Moderator analyses.

Moderator	*b*	Omnibustest	Test for residual heterogeneity	*k*	*N* ES
**Level 3**					
Study Quality	0.086	F(1, 53) = 3.138	QE (53) = 2066.045***	35	55
Country (high-income vs. not)	−0.065	F(1, 53) = 0.949	QE (53) = 2282.205***	35	55
Mean Age	−0.010	F(1, 51) = 0.801	QE (51) = 2236.129***	34	52
Single women in sample (%)	−0.003	F(1, 35) = 0.455	QE (35) = 280.736***	24	37
Primiparous women in sample (%)	−0.002	F(1, 44) = 2.023	QE (44) = 1504.409***	29	46
Vaginal birth non-instrumental (%)	0.002	F(1, 26) = 0.975	QE (26) = 1602.162***	19	28
Unplanned CS /instrumental birth (%)	−0.000	F(1, 26) = 0.001	QE (26) = 1328.665***	18	28
Planned CS (%)	−0.008*	F(1, 31) = 7.300	QE (31) = 585.345***	21	33
General level of intervention (high vs. low)	−0.004	F(1, 41) = 0.003	QE (41) = 1717.292***	28	43
**Level 2**					
Outcome measurement method (questionnaire vs. interview)	0.044	F(1, 47) = 0.444	QE (47) = 2201.281***	31	49
Number of items in outcome measurement	−0.005	F(1, 52) = 2.241	QE (52) = 1392.540***	34	54
Number of items in decision-making measurement	−0.003	F(1, 52) = 1.218	QE (52) = 2146.891***	34	54
Measurement time point (days after birth)	−0.045	F(1, 51) = 1.089	QE (51) = 396.552***	34	53

Note: *p < 0.05, **p < 0.01, ***p < 0.0001. *k* = number of independent studies. *N* ES = Number of effect sizes.

Second, all potential moderators were tested with respect to only PP-D outcomes and PP-PTSD outcomes respectively. For PP-D, two significant moderator effects were found. On Level 3 the dichotomous variable indicating whether the study was performed in a high-income country or not had a modifying impact, indicating that in middle – and low-income countries the correlation between decision-making and PP-D was stronger than in high-income countries (*b* = −0.170, *p* = 0.014, F[1, 16] = 7.579, *p* = 0.014, QE[16] = 85.205, *p* < 0.0001, *k* = 13, *N* ES = 18). On Level 2 a dummy variable showing whether the correlation was based on a single-item measure of decision-making or several-item scale turned out to be a significant moderator (*b* = −0.234, *p* = 0.002, F[1, 16] = 13.074, *p* = 0.002, QE[16] = 169.985, *p* < 0.0001, *k* = 13, *N* ES = 18). Correlations were stronger and significantly different from zero when multi-item scales where applied (*r* = −0.09, *p* = 0.002), which was not the case for single-item measurement (*r* = 0.14, *p* = 0.179).

For PP-PTSD, as also found for overall mental health, the amount of planned c-sections in the samples was a significant moderator (*b* = −0.221, *p* = 0.008, F[1, 17] = 9.094, *p* = 0.008, QE[17] = 135.036, *p* < 0.0001, *k* = 13, *N* ES = 19). On Level 2 the measurement time point significantly reduced heterogeneity among effect sizes within the studies (*b* = -.098, *p* = 0.0319, F[1, 26] = 5.143, *p* = 0.0319, QE[26] = 84.607, *p* < 0.0001, *k* = 20, *N* ES = 28). This means that the correlation was stronger in studies that had a greater time lag between birth and postnatal outcome measurement. When outcomes were measured less than 100 days after birth the mean effect was *r* = −0.13 (*p* = 0.050). Outcome measurement after this cut-off, resulted in a mean effect of *r* = −0.23 (*p* = 0.032), indicating that the manifestation of PP-PTSD after having experienced ineffective decision-making shows increasingly strong over time.

### Publication bias

**Figure 3.**
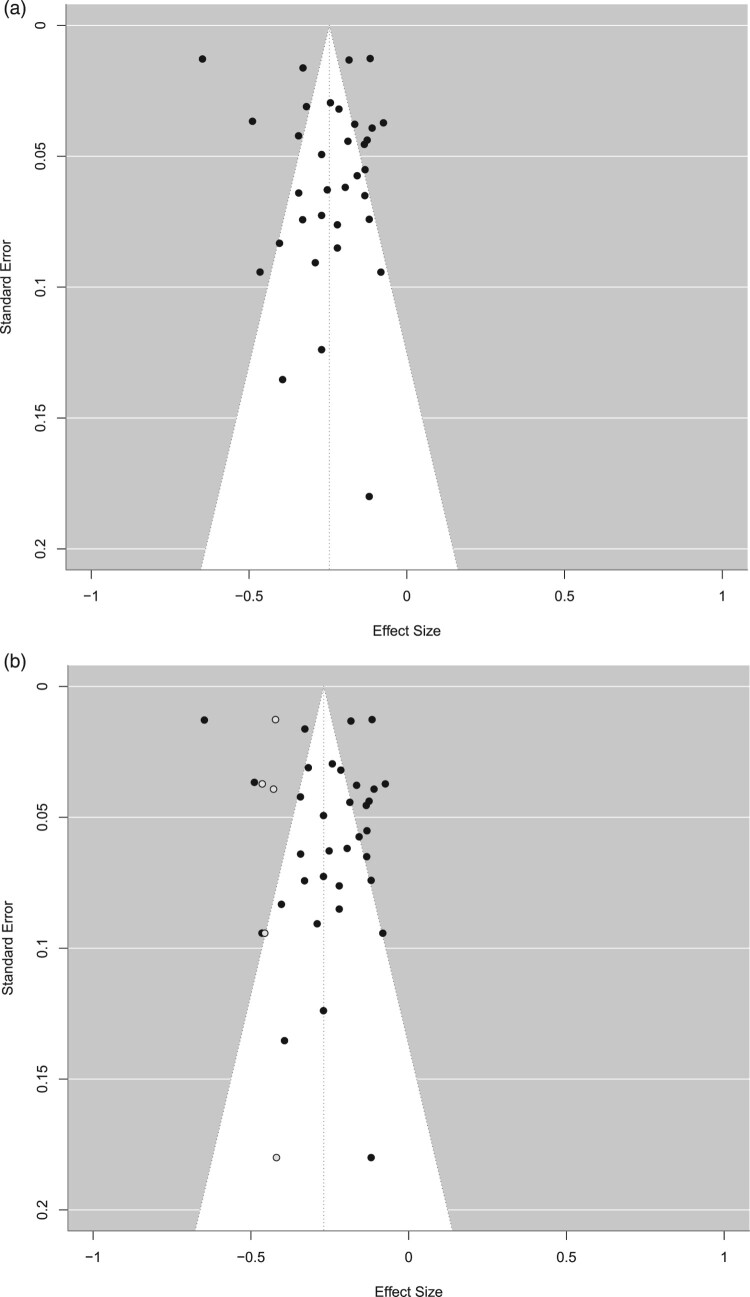
(a) Funnel plot of the association between decision-making and mental health outcomes. (b) Trim-and-fill funnel plot of the association between decision-making and mental health outcomes.

The test of funnel plot asymmetry was not significant (t (32) = 1.00, *p* = .326) (50), indicating that no publication bias is present (Figure 3(a)). A trim-and-fill analysis (Duval & Tweedie, 2000) performed with Lo estimators nevertheless reveiled 5 (white dots in Figure 3(b)) missing studies on the left side. Taking these studies into account, the adjusted effect was *r* = −0.27 (SE = 0.0233, CI = [−0.31; – 0.22], *p* < 0.0001). The results for the association of decision-making and PP-D-symptoms did not reveal any missing publications. The trim-and-fill analysis for the association of decision-making and PP-PTSD-symptoms estimated six missing studies on the left side of the funnel plot. This is indicative of missing publications with stronger negative effect sizes. Hence, the adjusted correlation between decision-making and PP-PTSD was estimated to be *r* = −0.34 (SE = 0.0324, CI = [−0.41; – 0.28], *p* < 0.0001).

## Conclusions

Our meta-analysis (*k* = 34, *N *= 34.586) investigated the association between intrapartum decision-making and postpartum mental health. Results revealed a significant negative correlation of moderate size (Gignac & Szodorai, 2016), which means that less effective decision-making goes along with a higher rate of psychopathology, which also held true for the two outcome groups PP-D and PP-PTSD. More precisely, while all four domains of decision-making were associated with overall mental health, only information, respect, and control were associated with PP-D, and only involvement, respect, and control were associated with PP-PTSD. This suggests that, besides respect and control, receiving a sufficient amount of information during birth is crucial for PP-D and being involved in decision-making during birth is essential for the development of PP-PTSD.

### Decision-making and overall postpartum mental health

The association of overall intrapartum decision-making and postpartum overall mental healt (*r* = −0.25) is consistent with previous research, highlighting the crucial role of birthing people’s relationship with care providers for postpartum adjustment (Stadlmayr et al., 2006) and social support as a protective factor for PTSD caused by interpersonal violence (Charuvastra & Cloitre, 2008). Notably, all four domains of decision-making during birth were associated with overall mental health, indicating that all are central for development postpartum mental health problems. These findings lend additional empirical support to the urgent call for respectful maternity care and reproductive justice (Kennedy et al., 2018; The White Ribbon Alliance, 2021; World Health Organization, 2015; World Health Organization, 2018). The results from moderator-analyses produced the interesting finding, that a higher rate of planned c-sections was related to a stronger relationship between decision-making and psychopathology. When there were more than 15% c-sections in a sample the correlation coefficient doubled. This leads to the assumption that experiencing poor decision-making in a seemingly 100% plannable birth setting can have an even more disturbing effect on post partum mental health, suggesting that, even in this setting respectful decision-making and involvement of the birthing person are crucial.

### Decision-making and PP-D

The magnitude of the association of between overall decision-making and PP-D (*r* = −0.19) is smaller than synthesized effect-sizes for PP-D and constructs similar to intrapartum decision-making in comparable meta-analyses, such as social support (*r* = −0.30), stressful life events (*r* = −0.29), violence experience (*r* = 0.32) (O'Hara & Swain, 1996; Wu et al., 2012). An explanation for these stronger associations might be the broadness of the used constructs. In contrast, research also revealed smaller synthesized associations between PP-D and birth-related variables, such as delivery complications (*r* = −0.13) or emergency c-sections (*r* = −0.11) (O'Hara & Swain, 1996; Xu et al., 2017). This underscores the powerful impact of interpersonal violence on severe mental health outcomes (as compared to events without interpersonal violence) (Cisler et al., 2012). However, the fact that the association found in our study is rather small is in line with research on the genesis of PP-D, attributing a more prominent role to psychological, social, and genetic factors (Guintivano et al., 2018), hormones (Amiel Castro et al., 2019; Brummelte & Galea, 2016), and more constant environmental and social factors than to acute events (Hahn-Holbrook et al., 2018). Nevertheless, our finding is indicative of a predictive value of ineffective decision-making for PP-D. Lastly, there was no evidence that being involved is associated with PP-D, suggesting that a birthing person’s level of involvement plays no role in the etiology of PP-D.

Moderator analyses revealed that a higher number of items measuring the ways of decision-making and having conducted the study in a low-income country were both related to an amplified correlation between decision-making and PP-D. For the former, a higher number of items is likely to result in a more reliable measurement with better predictive properties, which may cause the stronger association. For the latter, people from low-income countries might be more vulnerable due to existing stressors (e.g. food insecurity, lack of social security coverage, precarious job situation) and consequently have a higher risk for PP-D, emphasising the consideration of intersecting vulnerabilities also in the intrapartum period (Bohren et al., 2024; Santana et al., 2021).

### Decision-making and PP-PTSD

The association between overall decision-making and PP-PTSD (*r* = −0.29) is consistent with previous research, revealing an association between birthing-person-provider-interactions and PTSD-symptoms (Simpson & Catling, 2016). The magnitude of the association in our study, however, is smaller than synthesized effects for PP-PTSD and intrapartum support (*r* = −0.38), a negative subjective birth experience (*r* = 0.59), or perceived quality of interactions with staff (*r* = −0.40), (Ayers et al., 2016; Grekin & O'Hara, 2014). Again, the relative broadness of the used constructs may explain the stronger associations. In contrast, research also revealed smaller synthesized associations between PP-PTSD and birth-related variables, such as complications (*r* = 0.26) or intrapartum pain (*r* = 0.24) (Grekin & O'Hara, 2014). Remarkably, it appears that external control (e.g. over decisions, performed procedures) is at least an equally strong predictor of PP-PTSD as an internal control (e.g. over body, emotions) (Ford & Wright, 2009). This is not surprising, since interpersonal traumatic events are more likely to result in PTSD than other types of traumatic events (Charuvastra & Cloitre, 2008). It has to be noted that the trim-and-fill analysis indicated missing publications with even stronger negative effect sizes. In sum, our finding is indicative of a predictive value of ineffective decision-making for PP-PTSD. Finally, there was no evidence that being informed is associated with PP-PTSD, suggesting that receiving the expected amount of information plays no role in the etiology of PP-PTSD.

Later post-birth measurements, and planned c-sections were significant moderators and amplified the relationship between decision-making and PP-PTSD. The moderating effect of a later measurement time point can be attributed to a delayed effect of decision-making on PP-PTSD, which is both consistent (Stadlmayr et al., 2006) and inconsistent (Ayers et al., 2016) with similar research and thus warrants further examination. Lastly, another explanation for the moderating effect of planned c-sections could be that they are preceded by relationship-building through antenatal contacts and thus the experience of ineffective decision-making during surgery in these cases may be more unexpected and traumatic than in cases with less pre-established rapport between birthing people and providers, ultimately causing stronger associations. Alternatively, birthing people with planned c-sections may benefit less from the protective function of hormones and birthing consciousness and hence effects of ineffective decision-making may be more severe (Dahan, 2021; Kenkel, 2021). Finally, it has to be noted that unplanned c-sections did not moderate the association between overall decision-making and PP-D.

### Limitations

Most included studies were of moderate or low study quality. Only ten studies provided measurements of pre-birth psychological distress and, thus, respective moderator analysis was limited. Only two studies measured psychological outcomes later than one year after birth, which rendered synthesising long-term effects of decision-making impossible. The great majority of studies stemmed from high-income countries; hence, generalizability of our findings is limited. Lastly, heterogeneity in intrapartum decision-making measures might limit interpretability of results concerning the extracted four domains of decision-making during birth. Based on the analyses of publication bias, it can be concluded that available research on PP-PTSD as an outcome might be biased due to publication strategies. The analyses suggested, that especially studies with more pronounced negative effects might be missing. Future research and publication policies should address this gap.

## Conclusion

Our study evidences that ineffective intrapartum decision-making contributes to the genesis postpartum psychopathology. Interestingly, this finding marks a convergence of ethico-legal and psychological aspects, as rights-based care (materialising in the form of effective decision-making) is not only a normative imperative but translates into better health. Moreover, our nuanced findings on the relationships between the four dimensions of decision-making and the two psychological outcomes (PP-D, PP-PTSD) provide tangible hints as to how to shape more effective and healthy decision-making during birth (Rost et al., 2022; Rost et al., 2023). Given the high number of births, the widespread occurrence of ineffective intrapartum decision-making, the resulting mental health problems and associated costs, our findings are also relevant from a public health and health economics perspective. However, more research is needed to substantiate the various pathways through which the dimensions of decision-making can translate into adverse psychological outcomes and to draw more robust conclusions about causality, for example, through randomized intrapartum decision-making intervention studies or study designs allowing to control for pre-birth psychological distress.

Future meta-analyses will benefit from more and more widely applied validated instruments measuring decision-making (Vedam et al., 2017; Vedam et al., 2017). In line with the WHO, main implications for practice concern establishing numerous antenatal care contacts as a standard to enhance both birthing people’s and providers’ birth preparedness, and measuring the experience of decision-making as an indicator of quality of intrapartum care as a default to monitor, analyse, and improve decision-making as well as to facilitate accountability systems (World Health Organization, 2014; World Health Organization, 2016). More effective decision-making will translate into better health of parents and children. Finally, it is time to universally acknowledge that ineffective decision-making does cause psychological harm on the part of birthing people and to collaboratively work towards effectuating fundamental change (Rost et al., 2020).

## Authors’ contribution

**Table:** 

	1st author	2nd author	3rd author	4th author
Conceptualization	x			x
Data curation	x	x		x
Formal Analysis	x	x		x
Funding acquisition				x
Investigation	x			x
Methodology	x	x	x	x
Project administration				x
Resources				x
Software	x			x
Supervision			x	x
Validation			x	x
Visualisation	x			x
Writing – original draft	x	x		x
Writing – review & editing	x	x	x	x

## Data Availability

All data used in this meta-analysis were obtained from previously published studies. A full list of the included studies is available upon request from the corresponding author.

## Appendix

**Table A1.** Search terms.

		Matches					
	Search terms	Scopus	Pub Med	Psyc Info	Web of Science	Cinahl	Soc Index
1)	Consent OR decision* OR rapport OR autonomy OR communicat* OR choice OR self-determin*	5.223.405	1.132.651	563.353	1.964.984	574.005	230.315
2)	Birth* OR childbirth OR ‘maternity care’ OR intrapartum OR perinatal OR obstetric* OR delivery	1.909.118	954.520	131.156	822.370	372.947	49.828
3)	Psychological OR mental OR emotional OR depression OR anxiety OR stress OR trauma* OR ‘well-being’ OR psychosis OR mood	6.020.061	2.266.565	1.355.122	2.659.691	969.118	245.083
4)	Postpartum OR post-partum OR ‘after childbirth’ OR ‘after birth’ OR ‘postnatal’ OR ‘post-natal’	280.058	218.829	36.042	167.482	61.291	3.466
5)	1) AND 2) AND 3) AND 4)	2.565	1.102	478	967	995	56
	Total: 6.163						
Date of last search: 16.12.2021

**Table A2.** Scales and single items measuring intrapartum decision-making.

Scale	Description	α	Used by	Domain of Decision-making
BEI: Birth experience interview (Kountanis et al., 2021)	Semi-structured interview quantified through coding of subjective perception of communication (positive vs. mixed and negative)	N/A	Kountanis, Kirk (Kountanis et al., 2021)	Being involved
DDMS: Delivery Decision-Making Scale (Attanasio et al., 2018)	6 items, shared decision-making	.69	Kjerulff, Attanasio (Kjerulff et al., 2021)	Being involved
MADM: Mother’s autonomy in decision making scale (Vedam et al., 2017)	7 items, birthing persons’ autonomy in decision-making	.96	Limmer, Stoll (Limmer et al., 2021)	Being involved
QPI-I: quality of provider interactions inventory (Sorenson & Tschetter, 2010)	29 items, providers’ interpersonal relationship behaviors	.96	Sorenson and Tschetter (Sorenson & Tschetter, 2010)	Being involved
MOR-7: Mothers on respect index (Vedam et al., 2017)	7 items, respect for the birthing person and their preferences	.95	Limmer, Stoll (Limmer et al., 2021)	Being respected
MOR-G: Mothers on respect index (Vedam et al., 2017)	13 items, respect for the birthing person and their preferences	.95	Limmer, Stoll (Limmer et al., 2021)	Being respected
PCACL-R: Perceptions of Care Adjective Checklist – Revised (Redshaw & Martin, 2009)	16 items, positive and negative adjectives describing perceptions of care	N/A	Michels, Kruske (Michels et al., 2013)	Being respected
PCMC: person-centred maternity care scale (Afulani et al., 2017)	30 items, person-centredness of care	.84	Sudhinaraset, Landrian (Sudhinaraset et al., 2021)	Being respected
PCQ: Perception of Care Questionnaire (Fisher, 1994)	8 items technical subscale, 6 items emotional subscale	.90	Creedy, Shochet (et al., 2000)	Being respected
PCQ: Perception of Care Questionnaire (Fisher, 1994)	22 items, technical skills, decision-making, gentleness	.90	Verreault, Da Costa (Verreault et al., 2012)	Being respected
SCIB: support and control in birth questionnaire (Ford et al., 2009)	12 items, support subscale	.90	Ayers, Jessop (Ayers et al., 2014); Ford and Ayers (Ford & Ayers, 2011); Gökçe İsbİr, İncİ (Gökçe İsbİr et al., 2016); Tomsis, Perez (Tomsis et al., 2021)	Being respected
VPN: violence per negligence (Souza et al., 2017)	3 items, negligence of healthcare professionals	N/A	Souza, Rattner (Souza et al., 2017)	Being respected
Informal coercion (Oelhafen et al., 2021)	7 items, informed consent, opposition to intervention, intimidation, manipulation	N/A	Oelhafen, Trachsel (Oelhafen et al., 2021)	Being respected
EC2: Experience of control during childbirth scale (DeLuca & Lobel, 2014)	21 items, perceived control during childbirth	.89	DeLuca and Lobel (DeLuca & Lobel, 2014)	Having control
LADSI: labor and delivery satisfaction index, 6 Items from the birth satisfaction scale (Lomas et al., 1987)	6 items, birth satisfaction concerning control	.93	Fair and Morrison (Fair & Morrison, 2012)	Having control
LAS: Labor agentry Scale (Hodnett & Simmons-Tropea, 1987)	14 items, external control subscale	N/A	Denis, Parantv(Denis et al., 2011)	Having control
PCCh: Perceived control in childbirth scale (Stevens et al., 2012)	12 items, perceived control	.91	Stevens, Wallston (Stevens et al., 2012)	Having control
PCON: Perceived Control Scale (Wallston, 1989)	6 items, perceived control	N/A	Czarnocka and Slade (Czarnocka & Slade, 2000)	Having control
SCIB: support and control in birth questionnaire (Ford et al., 2009)	11 items, external control subscale	.86	Ford and Ayers (Ford & Ayers, 2011); Gökçe İsbİr, İncİ (Gökçe İsbİr et al., 2016); Tomsis, Perez (Tomsis et al., 2021)	Having control
BES: Birth Experiences Scale (Ayers, 1999)	3 items, subscale control over analgesia	.71	Keogh, Ayers (Keogh et al., 2002)	Having control
CEQ: Childbirth Experience Questionnaire (Dencker et al., 2010)	3 items, subscale participation	.80	King, McKenzie-McHarg (King et al., 2017); Avignon, David (Avignon et al., 2022)	Having control

**Table:**

Single Items	Used by	Domain of Decision-making
Not always kept informed	Mohammad, Gamble (Mohammad et al., 2011)	Being informed
Wanted more information during labor	Mohammad, Gamble (Mohammad et al., 2011)	Being informed
Wanted more information about why induction was necessary	Mohammad, Gamble (Mohammad et al., 2011)	Being informed
Overall rating of feeling informed	Leeds and Hargreaves (Leeds & Hargreaves, 2008)	Being informed
Quality of information given (adequate/inadequate)	Soet, Brack (Soet et al., 2003)	Being informed
Midwifes and doctors talked to me in a way I could understand	Redshaw and Henderson (Redshaw & Henderson, 2013)	Being informed
Information from doctors/midwives (enough/ would have like more)	Astbury, Brown (Astbury et al., 1994)	Being informed
Decisions made without taking my wishes into account	Mohammad, Gamble (Mohammad et al., 2011)	Being involved
Wishes listened to by staff	Leeds and Hargreaves (Leeds & Hargreaves, 2008)	Being involved
Involved in nonemergency decisions	Green, Coupland (Green et al., 1990)	Being involved
Allowed to play active role	Czarnocka and Slade (Czarnocka & Slade, 2000)	Being involved
Degree wishes and views were listened to by staff	Czarnocka and Slade (Czarnocka & Slade, 2000)	Being involved
Getting questions answered	Czarnocka and Slade (Czarnocka & Slade, 2000)	Being involved
Did not have active say in decision making about care in labour, all, or most of the time	Small, Lumley (Small et al., 2003)	Being involved
Active say in decision-making	Astbury, Brown (Astbury et al., 1994)	Being involved
Discussion of pain relief	Astbury, Brown (Astbury et al., 1994)	Being involved
Possibility to ask questions	De Schepper, Vercauteren (De Schepper et al., 2016)	Being involved
Staff communicated well	Henderson and Redshaw (Henderson & Redshaw, 2013)	Being involved
Respect	Redshaw and Henderson (Redshaw & Henderson, 2013)	Being respected
Birth plan (no; yes but not respected; yes and was respected)	Martinez-Vázquez, Rodríguez-Almagro (Martinez-Vázquez et al., 2021)	Being respected
Felt pressure to have baby quickly	Mohammad, Gamble (Mohammad et al., 2011)	Being respected
Pressure to have induction of labor	C. T. Beck, Gable (Beck et al., 2011)	Being respected
Pressure to have epidural analgesia	C. T. Beck, Gable (Beck et al., 2011)	Being respected
Felt labor was taken over by strangers/machines	Mohammad, Gamble (Mohammad et al., 2011)	Having control
Felt out of control	Mohammad, Gamble (Mohammad et al., 2011)	Having control
Control regarding the staff	Green, Coupland (Green et al., 1990)	Having control
In general, did you feel in control of what the staff were doing to you during labor	Green and Baston (Green & Baston, 2003)	Having control
Feeling in control of situation in labor	Czarnocka and Slade (Czarnocka & Slade, 2000)	Having control
Feelings of control/powerlessness	Soet, Brack (Soet et al., 2003)	Having control

## References

[CIT0001] Afulani, P. A., Diamond-Smith, N., Golub, G., & Sudhinaraset, M. (2017). Development of a tool to measure person-centered maternity care in developing settings: Validation in a rural and urban Kenyan population. *Reproductive Health*, *14*(1), 1–18. 10.1186/s12978-016-0263-4 28938885 PMC5610540

[CIT0002] Amiel Castro, R. T., Pataky, E. A., & Ehlert, U. (2019). Associations between premenstrual syndrome and postpartum depression: A systematic literature review. *Biological Psychology*, *147*, 107612. 10.1016/j.biopsycho.2018.10.014 30452945

[CIT0003] Ansari, H., & Yeravdekar, R. (2020). Respectful maternity care during childbirth in India: A systematic review and meta-analysis. *Journal of Postgraduate Medicine*, *66*(3), 133–140. 10.4103/jpgm.JPGM_648_19 32675449 PMC7542060

[CIT0004] Assink, M., & Wibbelink, C. J. M. (2016). Fitting three-level meta-analytic models in R: A step-by-step tutorial. *TQMP*, *12*(3), 154–174. 10.20982/tqmp.12.3.p154

[CIT0005] Astbury, J., Brown, S., Lumley, J., & Small, R. (1994). Birth events, birth experiences and social differences in postnatal depression. *Australian Journal of Public Health*, *18*(2), 176–184. 10.1111/j.1753-6405.1994.tb00222.x 7948335

[CIT0006] Attanasio, L. B., Kozhimannil, K. B., & Kjerulff, K. H. (2018). Factors influencing women’s perceptions of shared decision making during labor and delivery: Results from a large-scale cohort study of first childbirth. *Patient Education and Counseling*, *101*(6), 1130–1136. 10.1016/j.pec.2018.01.002 29339041 PMC5977392

[CIT0007] Avignon, V., David, B., Laurent, G., Corinne, D., & Horsch, A. (2022). Childbirth experience, risk of PTSD and obstetric and neonatal outcomes according to antenatal classes attendance. *Scientific Reports*, *12*, 10717. 35739298 10.1038/s41598-022-14508-z PMC9225805

[CIT0008] Ayers, S. (1999). *Post-traumatic stress disorder following childbirth: St George's*. University of London.

[CIT0009] Ayers, S., Bond, R., Bertullies, S., & Wijma, K. (2016). The aetiology of post-traumatic stress following childbirth: A meta-analysis and theoretical framework. *Psychological Medicine*, *46*(6), 1121–1134. 10.1017/S0033291715002706 26878223

[CIT0010] Ayers, S., Jessop, D., Pike, A., Parfitt, Y., & Ford, E. (2014). The role of adult attachment style, birth intervention and support in posttraumatic stress after childbirth: A prospective study. *Journal of Affective Disorders*, *155*, 295–298. 10.1016/j.jad.2013.10.022 24238870

[CIT0011] Batt, M. M., Duffy, K. A., Novick, A. M., Metcalf, C. A., & Epperson, C. N. (2020). Is postpartum depression different from depression occurring outside of the perinatal period? A review of the evidence. *Focus (American Psychiatric Publishing)*, *18*(2), 106–119. 33162848 10.1176/appi.focus.20190045 PMC7587887

[CIT0012] Beck, C. T., Gable, R. K., Sakala, C., & Declercq, E. R. (2011). Posttraumatic stress disorder in new mothers: Results from a two-stage U. S. National Survey. *Birth*, *38*(3), 216–227. 10.1111/j.1523-536X.2011.00475.x 21884230

[CIT0013] Birth Place Lab. (2022). Person-centred decision making – key elements Vancouver, Canada: University of British Columbia. https://www.birthplacelab.org/wp-content/uploads/2020/05/Person-Centred-Decision-Making.pdf

[CIT0014] Bohren, M. A., Iyer, A., Barros, A. J. D., Williams, C. R., Hazfiarini, A., Arroyave, L., Filippi, V., Chamberlaing, C., Kabakian-Khasholianh, T., Mayra, K., Gill, R. Vogel, J., Choum, D., George, A. S., & Oladapo, O. T. (2024). Towards a better tomorrow: Addressing intersectional gender power relations to eradicate inequities in maternal health. *eClinicalMedicine*, *67*. 10.1016/j.eclinm.2023.102180 PMC10837533 38314054

[CIT0015] Bohren, M. A., Mehrtash, H., Fawole, B., Maung, T. M., Balde, M. D., Maya, E., Thwin, S. S., Aderoba, A. K., Vogel, J. P., Irinyenikan, T. K., Adeyanju, A. O., Mon, N. O., Adu-Bonsaffoh, K., Landoulsi, S., Guure, C., Adanu, R., Diallo, B. A., Gülmezoglu, A. M., Soumah, A. M., ... Tuncalp, Ö. (2019). How women are treated during facility-based childbirth in four countries: A cross-sectional study with labour observations and community-based surveys. *The Lancet*, *394*(10210), 1750–1763. 10.1016/S0140-6736(19)31992-0 PMC6853169 31604660

[CIT0016] Bohren, M. A., Vogel, J. P., Hunter, E. C., Lutsiv, O., Makh, S. K., Souza, J. P., Aguiar. C., Coneglian, F. S., Araújo Diniz, A. L., Tunçalp, Ö., Javadi, D., Oladapo, O. T., Khosla, R., Hindin, M. J., & Gülmezoglu, A. M. (2015). The mistreatment of women during childbirth in health facilities globally: A mixed-methods systematic review. *PLoS Medicine*, *12*(6), e1001847. 10.1371/journal.pmed.1001847 26126110 PMC4488322

[CIT0017] Brummelte, S., & Galea, L. A. M. (2016). Postpartum depression: Etiology, treatment and consequences for maternal care. *Hormones and Behavior*, *77*, 153–166. 10.1016/j.yhbeh.2015.08.008 26319224

[CIT0018] Callahan, J. L., Borja, S. E., & Hynan, M. T. (2006). Modification of the perinatal PTSD questionnaire to enhance clinical utility. *Journal of Perinatology: Official Journal of the California Perinatal Association*, *26*(9), 533–539. 16826190 10.1038/sj.jp.7211562

[CIT0019] Charuvastra, A., & Cloitre, M. (2008). Social bonds and posttraumatic stress disorder, Annual Review of Psychology, *59*(1), 301–328. 10.1146/annurev.psych.58.110405.085650 PMC2722782 17883334

[CIT0020] Cheung, M. W. (2014). Modeling dependent effect sizes with three-level meta-analyses: A structural equation modeling approach. *Psychological Methods*, *19*(2), 211–229. 10.1037/a0032968 23834422

[CIT0021] Cisler, J. M., Begle, A. M., Amstadter, A. B., Resnick, H. S., Danielson, C. K., Saunders, B. E., & Kilpatrick, D. G. (2012). Exposure to interpersonal violence and risk for PTSD, depression, delinquency, and binge drinking among adolescents: Data from the NSA-R. *Journal of Traumatic Stress*, *25*(1), 33–40. 10.1002/jts.21672 22354506 PMC4090767

[CIT0022] Cook, N., Ayers, S., & Horsch, A. (2018). Maternal posttraumatic stress disorder during the perinatal period and child outcomes: A systematic review. *Journal of Affective Disorders*, *225*, 18–31. 10.1016/j.jad.2017.07.045 28777972

[CIT0023] Cox, J. L., Holden, J. M., & Sagovsky, R. (1987). Detection of postnatal depression. Development of the 10-item Edinburgh Postnatal Depression Scale. *The British Journal of Psychiatry: The Journal of Mental Science*, *150*(6), 782–786. 3651732 10.1192/bjp.150.6.782

[CIT0024] Creedy, D. K., Shochet, I. M., & Horsfall, J. (2000). Childbirth and the development of acute trauma symptoms: Incidence and contributing factors. *Birth (berkeley, Calif)*, *27*(2), 104–111. 10.1046/j.1523-536x.2000.00104.x 11251488

[CIT0025] Creedy, D. K., Shochet, I. M., & Horsfall, J. (2000). Childbirth and the development of acute trauma symptoms: Incidence and contributing factors. *Birth (berkeley, Calif)*, *27*(2), 104–111. 10.1046/j.1523-536x.2000.00104.x 11251488

[CIT0026] Czarnocka, J., & Slade, P. (2000). Prevalence and predictors of post-traumatic stress symptoms following childbirth. *The British Journal of Clinical Psychology*, *39*(1), 35–51. 10.1348/014466500163095 10789027

[CIT0027] Czarnocka, J., & Slade, P. (2000). Prevalence and predictors of post-traumatic stress symptoms following childbirth. *British Journal of Clinical Psychology*, *39*(1), 35–51. 10.1348/014466500163095 10789027

[CIT0028] Dahan, O. (2021). Obstetrics at odds with evolution: The consequences of interrupting adaptive birthing consciousness. *New Ideas in Psychology*, *63*, 100903. 10.1016/j.newideapsych.2021.100903

[CIT0029] Dekel, S., Stuebe, C., & Dishy, G. (2017). Childbirth induced posttraumatic stress syndrome: A systematic review of prevalence and risk factors. *Frontiers in Psychology*, *8*(560). 10.3389/fpsyg.2017.00560 PMC5387093 28443054

[CIT0030] DeLuca, R. S., & Lobel, M. (2014). Diminished control and unmet expectations: Testing a model of adjustment to unplanned cesarean delivery. *Analyses of Social Issues and Public Policy*, *14*(1), 183–204. 10.1111/asap.12040

[CIT0031] DeLuca, R. S., & Lobel, M. (2014). Diminished control and unmet expectations: Testing a model of adjustment to unplanned cesarean delivery. *Analyses of Social Issues and Public Policy (ASAP*, *14*(1), 183–204. 10.1111/asap.12040

[CIT0032] Dencker, A., Taft, C., Bergqvist, L., Lilja, H., & Berg, M. (2010). Childbirth experience questionnaire (CEQ): development and evaluation of a multidimensional instrument. *BMC Pregnancy and Childbirth*, *10*(1), 1–8. 10.1186/1471-2393-10-81 21143961 PMC3008689

[CIT0033] Denis, A., Parant, O., & Callahan, S. (2011). Post-traumatic stress disorder related to birth: A prospective longitudinal study in a French population. *Journal of Reproductive and Infant Psychology*, *29*(2), 125–135. 10.1080/02646838.2010.513048

[CIT0034] De Schepper, S., Vercauteren, T., Tersago, J., Jacquemyn, Y., Raes, F., & Franck, E. (2016). Post-Traumatic stress disorder after childbirth and the influence of maternity team care during labour and birth: A cohort study. *Midwifery*, *32*, 87–92. 10.1016/j.midw.2015.08.010 26410818

[CIT0035] Dikmen Yildiz, P., Ayers, S., & Phillips, L. (2017). Factors associated with post-traumatic stress symptoms (PTSS) 4-6 weeks and 6 months after birth: A longitudinal population-based study. *Journal of Affective Disorders*, *221*, 238–245. 10.1016/j.jad.2017.06.049 28654849

[CIT0036] Duval, S., & Tweedie, R. (2000). Trim and fill: A simple funnel-plot-based method of testing and adjusting for publication bias in meta-analysis. *Biometrics*, *56*(2), 455–463. 10.1111/j.0006-341X.2000.00455.x 10877304

[CIT0037] Egger, M., Smith, G. D., Schneider, M., & Minder, C. (1997). Bias in meta-analysis detected by a simple, graphical test. *Bmj*, *315*(7109), 629–634. 10.1136/bmj.315.7109.629 9310563 PMC2127453

[CIT0038] Fair, C. D., & Morrison, T. E. (2012). The relationship between prenatal control, expectations, experienced control, and birth satisfaction among primiparous women. *Midwifery*, *28*(1), 39–44. 10.1016/j.midw.2010.10.013 21458895

[CIT0039] Fisher, J. (1994). Obstetric intrvention: psychological predictors and psychological consequences [Unpublished Doctoral Thesis]. University of Melbourne.

[CIT0040] Ford, A. (2011). Support during birth interacts with prior trauma and birth intervention to predict postnatal post-traumatic stress symptoms. *Psychology & Health*, *26*(12), 1553–1570. 10.1080/08870446.2010.533770 21598181

[CIT0041] Ford, E., & Ayers, S. (2011). Support during birth interacts with prior trauma and birth intervention to predict postnatal post-traumatic stress symptoms. *Psychology & Health*, *26*(12), 1553–1570. 10.1080/08870446.2010.533770 21598181

[CIT0042] Ford, E., Ayers, S., & Wright, D. B. (2009). Measurement of maternal perceptions of support and control in birth (SCIB). *Journal of Women's Health*, *18*(2), 245–252. 10.1089/jwh.2008.0882 19183096

[CIT0043] Ford, A. S., & Wright, D. B. (2009). Measurement of maternal perceptions of support and control in birth (SCIB). *Journal of Women's Health*, *18*(2), 245–252. 10.1089/jwh.2008.0882 19183096

[CIT0044] Garthus-Niegel, S., Horsch, A., Handtke, E., von Soest, T., Ayers, S., Weidner, K., Eberhard-Gran, M. (2018). The impact of postpartum posttraumatic stress and depression symptoms on couples’ relationship satisfaction: A population-based prospective study. *Frontiers in Psychology*, *9*. 10.3389/fpsyg.2018.01728 PMC6157399 30283380

[CIT0045] Garthus-Niegel, S., von Soest, T., Vollrath, M. E., & Eberhard-Gran, M. (2013). The impact of subjective birth experiences on post-traumatic stress symptoms: A longitudinal study. *Archives of Women's Mental Health*, *16*(1), 1–10. 10.1007/s00737-012-0301-3 22940723

[CIT0046] Gignac, G. E., & Szodorai, E. T. (2016). Effect size guidelines for individual differences researchers. *Personality and Individual Differences*, *102*, 74–78. 10.1016/j.paid.2016.06.069

[CIT0047] Gökçe İsbİr, G., İncİ, F., Bektaş, M., Dikmen Yıldız, P., & Ayers, S. (2016). Risk factors associated with post-traumatic stress symptoms following childbirth in Turkey. *Midwifery*, *41*, 96–103. 10.1016/j.midw.2016.07.016 27571774

[CIT0048] Gottvall, K., & Waldenström, U. (2002). Does a traumatic birth experience have an impact on future reproduction? *BJOG: An International Journal of Obstetrics and Gynaecology*, *109*(3), 254–260. 10.1111/j.1471-0528.2002.01200.x 11950179

[CIT0049] Green, J. M., & Baston, H. A. (2003). Feeling in control during labor: Concepts, correlates, and consequences. *Birth: Issues in Perinatal Care*, *30*(4), 235–247. 10.1046/j.1523-536X.2003.00253.x 14992154

[CIT0050] Green, J. M., Coupland, V. A., & Kitzinger, J. V. (1990). Expectations, experiences, and psychological outcomes of childbirth: A prospective study of 825 women. *Birth (berkeley, Calif)*, *17*(1), 15–24. 10.1111/j.1523-536X.1990.tb00004.x 2346576

[CIT0051] Grekin, R., & O'Hara, M. W. (2014). Prevalence and risk factors of postpartum posttraumatic stress disorder: A meta-analysis. *Clinical Psychology Review*, *34*(5), 389–401. 10.1016/j.cpr.2014.05.003 24952134

[CIT0052] Guintivano, J., Manuck, T., & Meltzer-Brody, S. (2018). Predictors of postpartum depression: A comprehensive review of the last decade of evidence. *Clinical Obstetrics and Gynecology*, *61*(3), 591–603. 10.1097/GRF.0000000000000368 29596076 PMC6059965

[CIT0053] Hahn-Holbrook, J., Cornwell-Hinrichs, T., & Anaya, I. (2018). Economic and health predictors of national postpartum depression prevalence: A systematic review, meta-analysis, and meta-regression of 291 studies from 56 countries. *Frontiers in Psychiatry*, *8*. 10.3389/fpsyt.2017.00248 PMC5799244 29449816

[CIT0054] Harrer, M., Cuijpers, P., & Ebert, D. (2019). Doing Meta-Analysis in R.

[CIT0055] Harris, R., & Ayers, S. (2012). What makes labour and birth traumatic? A survey of intrapartum ‘hotspots’. *Psychology & Health*, *27*(10), 1166–1177. 10.1080/08870446.2011.649755 22292475

[CIT0056] Henderson, J., & Redshaw, M. (2013). Who is well after childbirth? Factors Related to Positive Outcome. *Birth: Issues in Perinatal Care*, *40*(1), 1–9. 10.1111/birt.12022 24635418

[CIT0057] Higgins, J. P. T. Thompson S.G. (2002). Quantifying heterogeneity in a meta-analysis, *Statistics in Medicine 21*(11), 1539–1558. 10.1002/sim.1186 12111919

[CIT0058] Higgins, J. P., Thompson, S. G., Deeks, J. J., & Altman, D. G. (2003). Measuring inconsistency in meta-analyses. *BMJ (Clinical Research ed*, *327*(7414), 557–560. 10.1136/bmj.327.7414.557 PMC192859 12958120

[CIT0059] Hodnett, E. D., & Simmons-Tropea, D. A. (1987). The Labour Agentry Scale: Psychometric properties of an instrument measuring control during childbirth. *Research in Nursing & Health*, *10*(5), 301–310. 10.1002/nur.4770100503 3671777

[CIT0060] IntHout, J., Ioannidis, J. P. A., & Borm, G. F. (2014). The Hartung-Knapp-Sidik-Jonkman method for random effects meta-analysis is straightforward and considerably outperforms the standard DerSimonian-Laird method. *BMC Medical Research Methodology*, *14*(1), 25. 10.1186/1471-2288-14-25 24548571 PMC4015721

[CIT0061] Kenkel, W. (2021). Birth signalling hormones and the developmental consequences of caesarean delivery, *Journal of Neuroendocrinology 33*(1), e12912. 10.1111/jne.12912 PMC10590550 33145818

[CIT0062] Kennedy, H. P., Cheyney, M., Dahlen, H., Downe, S., Foureur, M., Homer, C., Jefford, E., McFadden, A., Michel-Schuldt, M., Sandall, J., Soltani, H., Speciale, A., Stevens, J., Vedam, S., & Renfrew, M. J. (2018). Asking different questions: A call to action for research to improve the quality of care for every woman, every child. *Women and Birth*, *31*(4). 10.1016/j.wombi.2018.06.011 29945773

[CIT0063] Keogh, E., Ayers, S., & Francis, H. (2002). Does anxiety sensitivity predict post-traumatic stress symptoms following childbirth? A preliminary report. *Cognitive Behaviour Therapy*, *31*(4), 145–155. 10.1080/165060702321138546

[CIT0064] King, L., McKenzie-McHarg, K., & Horsch, A. (2017). Testing a cognitive model to predict posttraumatic stress disorder following childbirth. *BMC Pregnancy and Childbirth*, *17*(1), 32. 10.1186/s12884-016-1194-3 28088194 PMC5237569

[CIT0065] Kjerulff, K. H., Attanasio, L. B., Sznajder, K. K., & Brubaker, L. H. (2021). A prospective cohort study of post-traumatic stress disorder and maternal-infant bonding after first childbirth. *Journal of Psychosomatic Research*, *144*(110424). 10.1016/j.jpsychores.2021.110424 PMC8101703 33756149

[CIT0066] Kountanis, J. A., Kirk, R., Handelzalts, J. E., Jester, J. M., Kirk, R., & Muzik, M. (2021). The associations of subjective appraisal of birth pain and provider-patient communication with postpartum-onset PTSD. *Archives of Women's Mental Health*, 171–180. 10.1007/s00737-021-01154-z 34250546

[CIT0067] Leeds, L., & Hargreaves, I. (2008). The psychological consequences of childbirth. *J Reprod Infant Psyc*, *26*(2), 108–122. 10.1080/02646830701688299

[CIT0068] Limmer, C., Stoll, K., Vedam, S., & Gross, M. (2021a). Measuring disrespect and abuse during childbirth in a high-resource country: development and validation of a German Self-Report Tool. Preprint. 10.1016/j.midw.2023.103809 37689053

[CIT0069] Limmer, C., Stoll, K., Vedam, S., Leinweber, J., & Gross, M. (2021b). Measuring disrespect and abuse during childbirth in a high-resource country: development and validation of a German Self-Report Tool. 10.1016/j.midw.2023.103809 37689053

[CIT0070] Lomas, J., Dore, S., Enkin, M., & Mitchell, A. (1987). The labor and delivery satisfaction index: The development and evaluation of a soft outcome measure. *Birth (berkeley, Calif )*, *14*(3), 125–129. 10.1111/j.1523-536X.1987.tb01472.x 3318853

[CIT0071] Martinez-Vázquez, S., Rodríguez-Almagro, J., Hernández-Martínez, A., Delgado-Rodríguez, M., & Martínez-Galiano, J. M. (2021). Obstetric factors associated with postpartum post-traumatic stress disorder after spontaneous vaginal birth. *Birth (berkeley, Calif)*, *48*(3), 406–415. 10.1111/birt.12550 33909303

[CIT0072] Martinez-Vázquez, S., Rodríguez-Almagro, J., Hernández-Martínez, A., Delgado-Rodríguez, M., & Martínez-Galiano, J. M. (2021). Obstetric factors associated with postpartum post-traumatic stress disorder after spontaneous vaginal birth. *Birth: Issues in Perinatal Care*, *48*(3), 406–415. 10.1111/birt.12550 33909303

[CIT0073] Menage, J. (1993). Post-traumatic stress disorder in women who have undergone obstetric and/or gynaecological procedures: A consecutive series of 30 cases of PTSD. *Journal of Reproductive and Infant Psychology*, *11*(4), 221–228. 10.1080/02646839308403222

[CIT0074] Michels, A., Kruske, S., & Thompson, R. (2013). Women’s postnatal psychological functioning: The role of satisfaction with intrapartum care and the birth experience. *Journal of Reproductive and Infant Psychology*, *31*(2), 172–182. 10.1080/02646838.2013.791921

[CIT0075] Mohammad, K. I., Gamble, J., & Creedy, D. K. (2011). Prevalence and factors associated with the development of antenatal and postnatal depression among Jordanian women. *Midwifery*, *27*(6), e238–ee45. 10.1016/j.midw.2010.10.008 21130548

[CIT0076] Mselle, L. T., Moland, K. M., Mvungi, A., Evjen-Olsen, B., & Kohi, T. W. (2013). Why give birth in health facility? Users’ and providers’ accounts of poor quality of birth care in Tanzania. *BMC Health Services Research*, *13*(1), 174. 10.1186/1472-6963-13-174 23663299 PMC3654954

[CIT0077] Niles, P. M., Stoll, K., Wang, J. J., Black, S., & Vedam, S. (2021). I fought my entire way": experiences of declining maternity care services in British Columbia. *PLoS One*, *16*(6), e0252645. 10.1371/journal.pone.0252645 34086795 PMC8177419

[CIT0078] Oelhafen, S., Trachsel, M., Monteverde, S., Raio, L., & Cignacco, E. (2021). Informal coercion during childbirth: Risk factors and prevalence estimates from a nationwide survey of women in Switzerland. *BMC Pregnancy and Childbirth*, *21*(1), 369. 10.1186/s12884-021-03826-1 33971841 PMC8112037

[CIT0079] Oelhafen, S., Trachsel, M., Monteverde, S., Raio, L., & Müller, E. C. (2020). Informal Coercion During Childbirth: Risk Factors and Prevalence Estimates from a Nationwide Survey among Women in Switzerland, .10.16.20212480.

[CIT0080] O'Hara, M. W., & Swain, A. M. (1996). Rates and risk of postpartum depression—a meta-analysis. *International Review of Psychiatry*, *8*(1), 37–54. 10.3109/09540269609037816

[CIT0081] Olde, E., van der Hart, O., Kleber, R., & van Son, M. (2006). Posttraumatic stress following childbirth: A review. *Clinical Psychology Review*, *26*(1), 1–16. 10.1016/j.cpr.2005.07.002 16176853

[CIT0082] Page, M. J., McKenzie, J. E., Bossuyt, P. M., Boutron, I., Hoffmann, T. C., Mulrow, C. D., Mulrow, C. D., Shamseer, L., Tetzlaff, J.M., Akl, E. A., Brennan, S., Chou, R., Glanville, J., Grimshaw, J. M., Hróbjartsson, A., Lalu, M., Li, T., Loder, E. W., McDonald, S., McGuiness, L. A., Stewart, L. ... Moher, D. (2021). *The PRISMA*, statement: an updated guideline for reporting systematic reviews 372, n71. 10.1136/bmj.n71 PMC8005924 33782057

[CIT0083] Parfitt, Y. M., & Ayers, S. (2009). The effect of post-natal symptoms of post-traumatic stress and depression on the couple's relationship and parent–baby bond. *Journal of Reproductive and Infant Psychology*, *27*(2), 127–142. 10.1080/02646830802350831

[CIT0084] Redshaw, M., & Henderson, J. (2013). From antenatal to postnatal depression: Associated factors and mitigating influences. *Journal of Women's Health*, *22*(6), 518–525. 10.1089/jwh.2012.4152 23751165

[CIT0085] Redshaw, M., & Martin, C. R. (2009). Validation of a perceptions of care adjective checklist. *Journal of Evaluation in Clinical Practice*, *15*(2), 281–288. 10.1111/j.1365-2753.2008.00995.x 19335485

[CIT0086] Rosenthal, R., Cooper, H., & Hedges, L. (1994). Parametric measures of effect size. *Handbook of Research Synthesis*, *621*(2), 231–244.

[CIT0087] Rost, M., Arnold, L., & De Clercq, E. (2020). *Boiling up the Problem of Violence” in Childbirth?* An Ethical Viewpoint on Medical Professional Responses to Women’s Reports of Mistreatment in Childbirth Ethik in der Medizin, 2.

[CIT0088] Rost, M., Stuerner, Z., Niles, P., & Arnold, L. (2022). “Real decision-making is hard to find” - Swiss perinatal care providers’ perceptions of and attitudes towards decision-making in birth: A qualitative study. *Social Science and Medicine - Qualitative Research in Health*, *2*.

[CIT0089] Rost MS, Z., Niles, P., & Arnold, L. (2023). Between “a lot of room for it” and “it doesn’t exist” - advancing and limiting factors of autonomy in birth as perceived by perinatal care providers: An interview study. *Birth*, *50*, 1068–1080. 10.1111/birt.12757 37593797

[CIT0090] Santana, C. L. A., Manfrinato, C. V., Souza, P. R. P., Marino, A., Condé, V. F., Stedefeldt, E., Tomita, L. Y., & do Carmo Franco, M. (2021). Psychological distress, ow-income, and socio-economic vulnerability in the COVID-19 pandemic. *Public Health*, *199*, 42–45. 10.1016/j.puhe.2021.08.016 34537575 PMC8390360

[CIT0091] Shay, L. A., & Lafata, J. E. (2015). Where is the evidence? A Systematic Review of Shared Decision Making and Patient Outcomes. *Medical Decision Making*, *35*(1), 114–131. 10.1177/0272989X14551638 25351843 PMC4270851

[CIT0092] Sidik, K., & Jonkman, J. N. (2005). Simple heterogeneity variance estimation for meta-analysis. *Journal of the Royal Statistical Society 54*(2), 367–384.

[CIT0093] Silan, V., Kant, S., Archana, S., Misra, P., & Rizwan, S. (2014). Determinants of underutilisation of free delivery services in an area with high institutional delivery rate: A qualitative study. *North American Journal of Medical Sciences*, *6*(7), 315–320. 10.4103/1947-2714.136906 25077079 PMC4114008

[CIT0094] Simpson, M., & Catling, C. (2016). Understanding psychological traumatic birth experiences: A literature review. *Women and Birth*, *29*(3), 203–207. 10.1016/j.wombi.2015.10.009 26563636

[CIT0095] Small, R., Lumley, J., & Yelland, J. (2003). Cross-cultural experiences of maternal depression: Associations and contributing factors for Vietnamese, Turkish and Filipino immigrant women in Victoria, Australia. *Ethnicity & Health*, *8*(3), 189–206. 10.1080/1355785032000136416 14577995

[CIT0096] Soet, J. E., Brack, G. A., & DiIorio, C. (2003). Prevalence and predictors of women's experience of psychological trauma during childbirth. *Birth (berkeley, Calif)*, *30*(1), 36–46. 10.1046/j.1523-536X.2003.00215.x 12581038

[CIT0097] Sorenson, D., & Tschetter, L. (2010). Prevalence of negative birth perception, disaffirmation, perinatal trauma symptoms, and depression Among postpartum women. *Perspectives in Psychiatric Care*, *46*(1), 14–25. 10.1111/j.1744-6163.2009.00234.x 20051075

[CIT0098] Souza, K. J., Rattner, D., & Gubert, M. B. (2017). Institutional violence and quality of service in obstetrics are associated with postpartum depression. *Revista de Saude Publica*, *51*(69). 10.1590/S1518-8787.2017051006549 PMC5510781 28746574

[CIT0099] Stadlmayr, W., Amsler, F., Lemola, S., Stein, S., Alt, M., Bürgin, D., Surbek, D., Bitzer, J. (2006). Memory of childbirth in the second year: The long-term effect of a negative birth experience and its modulation by the perceived intranatal relationship with caregivers. *Journal of Psychosomatic Obstetrics & Gynecology*, *27*(4), 211–224. 10.1080/01674820600804276 17225622

[CIT0100] Stevens, N. R., Wallston, K. A., & Hamilton, N. A. (2012). Perceived control and maternal satisfaction with childbirth: A measure development study. *Journal of Psychosomatic Obstetrics & Gynecology*, *33*(1), 15–24. 10.3109/0167482X.2011.652996 22304395

[CIT0101] Størksen, H. T., Garthus-Niegel, S., Vangen, S., & Eberhard-Gran, M. (2013). The impact of previous birth experiences on maternal fear of childbirth. *Acta Obstetricia et Gynecologica Scandinavica*, *92*(3), 318–324. 10.1111/aogs.12072 23278249

[CIT0102] Sudhinaraset, M., Afulani, P., Diamond-Smith, N., Bhattacharyya, S., Donnay, F., & Montagu, D. (2017). Advancing a conceptual model to improve maternal health quality: The person-centered care framework for reproductive health equity. *Gates Open Research*, *1*(1). 10.12688/gatesopenres.12756.1 PMC5764229 29355215

[CIT0103] Sudhinaraset, M., Landrian, A., Golub, G. M., Cotter, S. Y., & Afulani, P. A. (2021). Person-centered maternity care and postnatal health: Associations with maternal and newborn health outcomes. *AJOG Global Reports*, *1*(1), 100005. 10.1016/j.xagr.2021.100005 33889853 PMC8041064

[CIT0104] Suetsugu, Y., Haruna, M., & Kamibeppu, K. (2020). A longitudinal study of bonding failure related to aspects of posttraumatic stress symptoms after childbirth among Japanese mothers. *BMC Pregnancy and Childbirth*, *20*(1), 434. 10.1186/s12884-020-03099-0 32727570 PMC7389449

[CIT0105] Thomas, B., Ciliska, D., Dobbins, M., & Micucci, S. (1998). Quality assessment tool for quantitative studies. The effective public health practice project (EPHPP): Effective Public Healthcare Panacea Project. https://www.ephpp.ca/quality-assessment-tool-for-quantitative-studies/

[CIT0106] Tomsis, Y., Perez, E., Sharabi, L., Shaked, M., Haze, S., & Hadid, S. (2021). Postpartum post-traumatic stress symptoms following cesarean section—the mediating effect of sense of control. *Psychiatric Quarterly*, *92*, 1839–1853. 34491482 10.1007/s11126-021-09949-0

[CIT0107] van der Pijl, M. S. G., Hollander, M. H., van der Linden, T., Verweij, R., Holten, L., Kingma, E., de Jonge, A., & Verhoeven, C. J. M. (2020). Left powerless: A qualitative social media content analysis of the Dutch #breakthesilence campaign on negative and traumatic experiences of labour and birth. *PLoS One*, *15*(5), e0233114. 32396552 10.1371/journal.pone.0233114 PMC7217465

[CIT0108] Vedam, S., Stoll, K., Martin, K., Rubashkin, N., Partridge, S., Thordarson, D., Thordarson, D., Jolicoeur, G., & the Changing Childbirth in BC Steering Council. (2017). The mother's autonomy in decision making (MADM) scale: Patient-led development and psychometric testing of a new instrument to evaluate experience of maternity care. *PLoS One*, *12*(2), e0171804. 10.1371/journal.pone.0171804 28231285 PMC5322919

[CIT0109] Vedam, S., Stoll, K., McRae, D. N., Korchinski, M., Velasquez, R., Wang, J., J., Partridge, S., McRae, L., Elwood Martin, R., Jolicoeur, G., & CCinBC Steering Committee (2019). Patient-led decision making: Measuring autonomy and respect in Canadian maternity care. *Patient Education and Counseling*, *102*(3), 586–594. 10.1016/j.pec.2018.10.023 30448044

[CIT0110] Vedam, S., Stoll, K., Rubashkin, N., Martin, K., Miller-Vedam, Z., Hayes-Klein, H., Jolicoeur, G., & the CCinBC Steering Council (2017). The mothers on respect (MOR) index: Measuring quality, safety, and human rights in childbirth. *SSM - Population Health*, *3*, 201–210. 10.1016/j.ssmph.2017.01.005 29349217 PMC5768993

[CIT0111] Vedam, S., Stoll, K., Taiwo, T. K., Rubashkin, N., Cheyney, M., Strauss, N., N., McLemore, M., Cadena, M., Nethery, E., Rushton, E., Schummers, L., Declercq E., & the GVtM-US Steering Council (2019). The giving voice to mothers study: Inequity and mistreatment during pregnancy and childbirth in the United States. *Reproductive Health*, *16*(1), 77. 10.1186/s12978-019-0729-2 31182118 PMC6558766

[CIT0112] Verreault, N., Da Costa, D., Marchand, A., Ireland, K., Banack, H., Dritsa, M., & Khalife, M. (2012). PTSD following childbirth: A prospective study of incidence and risk factors in Canadian women. *Journal of Psychosomatic Research*, *73*(4), 257–263. 10.1016/j.jpsychores.2012.07.010 22980529

[CIT0113] Viechtbauer, W. (2010). Conducting meta-analyses in R with the metafor package. *Journal of Statistical Software*, *36*(3), 1–48. 10.18637/jss.v036.i03

[CIT0114] Viera, A. J., & Garrett, J. M. (2005). Understanding interobserver agreement: The kappa statistic. *Family Medicine*, *37*(5), 360–363. 15883903

[CIT0115] Wallston, K. A. (1989). Assessment of control in health-care settings. *Stress, Personal Control and Health*, *1*, 85–105.

[CIT0116] The White Ribbon Alliance. (2021). Respectful Maternity Care Charter 2021 [Available from: https://www.whiteribbonalliance.org/respectful-maternity-care-charter/

[CIT0117] World Bank. (2022). New World Bank country classifications by income level: 2022-2023 2022 [Available from: https://blogs.worldbank.org/opendata/new-world-bank-country-classifications-income-level-2022-2023

[CIT0118] World Health Organization. (2014). The prevention and elimination of disrespect and abuse during facilitybased childbirth, WHO statement Geneva, Switzerland: World Health Organization.

[CIT0119] World Health Organization. (2015). Prevention and elimination of disrespect and abuse during childbirth. WHO statement. Geneva, Switzerland: WHO. https://apps.who.int/iris/bitstream/handle/10665/134588/WHO_RHR_14.23_eng.pdf

[CIT0120] World Health Organization. (2016). Standards for Improving Quality of Maternal and Newborn Care in Health Facilities: WHO. https://apps.who.int/iris/rest/bitstreams/1055891/retrieve

[CIT0121] World Health Organization. (2016). WHO recommendations on antenatal care for a positive pregnancy experience Geneva. Switzerland: WHO. Available from https://www.who.int/publications/i/item/9789241549912 28079998

[CIT0122] World Health Organization. (2018). *WHO recommendations: Intrapartum care for a positive childbirth experience Geneva*. 30070803

[CIT0123] Wu, Q., Chen, H.-L., & Xu, X.-J. (2012). Violence as a risk factor for postpartum depression in mothers: A meta-analysis. *Archives of Women's Mental Health*, *15*(2), 107–114. 10.1007/s00737-011-0248-9 22382278

[CIT0124] Xu, H., Ding, Y., Ma, Y., Xin, X., & Zhang, D. (2017). Cesarean section and risk of postpartum depression: A meta-analysis. *Journal of Psychosomatic Research*, *97*, 118–126. 10.1016/j.jpsychores.2017.04.016 28606491

